# Recanting of Previous Reports of Alcohol Consumption within a Large-Scale Clustered Randomised Control Trial

**DOI:** 10.1007/s11121-019-0981-2

**Published:** 2019-01-14

**Authors:** Andrew Percy, Ashley Agus, Jon Cole, Paul Doherty, David Foxcroft, Séamus Harvey, Michael McKay, Lynn Murphy, Harry Sumnall

**Affiliations:** 1 Centre for Evidence and Social Innovation, School of Social Sciences, Education and Social Work, Queen’s University Belfast, Belfast, UK; 2 Northern Ireland Clinical Trials Unit, Belfast, UK; 3 University of Liverpool, Liverpool, UK; 4 Oxford Brookes University, Oxford, UK; 5 Bangor University, Bangor, UK; 6 Liverpool John Moores University, Liverpool, UK

**Keywords:** Recanting, Measurement error, Alcohol, Self-report, RCT, prevention trial

## Abstract

The aim of this study was to examine the extent of recanting (inconsistencies in reporting of lifetime alcohol use) and its impact on the assessment of primary outcomes within a large-scale alcohol prevention trial. One hundred and five post-primary schools in were randomised to receive either the intervention or education as normal. Participants (*N* = 12,738) were secondary school students in year 8/S1 (mean age 12.5) at baseline. Self-report questionnaires were administered at baseline (T0) and at T1 (+ 12 months post-baseline), T2 (+ 24 months) and T3 (+ 33 months). The primary outcomes were (i) heavy episodic drinking (consumption of ≥ 6 units in a single episode in the previous 30 days for males and ≥ 4.5 units for females) assessed at T3 and (ii) the number of alcohol-related harms experienced in the last 6 months assessed at T3. Recanting was defined as a negative report of lifetime alcohol consumption that contradicted a prior positive report. Between T1 and T3, 9.9% of students recanted earlier alcohol consumption. Recanting ranged from 4.5 to 5.3% across individual data sweeps. While recanting was significantly associated (negatively) with both primary outcomes, the difference in the rate of recanting across trial arms was small, and adjusting for recanting within the primary outcome models did not impact on the primary outcome effects. Males were observed to recant at a greater rate than females, with a borderline small-sized effect (*V* = .09). While differential rates of recanting have the potential to undermine the analysis of prevention trial outcomes, recanting is easy to identify and control for within trial primary outcome analyses. Adjusting for recanting should be considered as an additional sensitivity test within prevention trials.

Trial Registration: ISRCTN47028486 (http://www.isrctn.com/ISRCTN47028486). The date of trial registration was 23/09/2011, and school recruitment began 01/11/2011.

Recanting, defined as the denial of a previous positive report of lifetime (ever used) substance use at a later interview, has been found to be a significant source of measurement error in longitudinal alcohol and drug-use surveys (Fendrich 2005; Fendrich and Rosenbaum 2003; Johnston and O'Malley 1997; Percy et al. 2005). Recanting can be identified through observing logical inconsistencies in the patterns of reporting of lifetime consumption across survey sweeps; as once a respondent has transitioned into substance use, all future responses to an ‘ever use’ question should be positive (Percy et al. 2005).

Recanting always indicates the presence of a false or mistaken report. It may arise from either over-reporting lifetime use (claiming to have tried alcohol when they actually had not, possibly to impress their peers) that is corrected at a later follow-up, or from under-reporting lifetime use at the later sweep (denying that they had ever used following an earlier positive report, typically due to social desirability or concerns about being identified as a drinker by either school or parents). Recanting may also be the result of a simple error, such as ticking the wrong box. Numerous longitudinal studies of adolescent alcohol and drug use have identified extensive recanting (Ensminger et al. 2007; Fendrich and Mackesy-Amiti 1995; Fendrich and Rosenbaum 2003; Johnston and O'Malley 1997; Percy et al. 2005; Shillington and Clapp 2000; Shillington et al. 2011a; Shillington et al. 1995; Shillington et al. 2011b; Taylor et al. 2017). Recanting has also been observed in relation to smoking (Sargent et al. 2017; Soulakova and Crockett 2014; Stanton et al. 2007), inhalant use (Martino et al. 2009), delinquency (Sibley et al. 2010), sexual behaviour (Dariotis et al. 2009; Palen et al. 2008), self-harm (Mars et al. 2016), weight control (Rosenbaum 2009) and age of substance use initiation (Bailey et al. 1992; Engels et al. 1997). Inconsistencies in self-reported substance use have not only been found across time but also across survey settings (school vs household) (Griesler et al. 2008).

In alcohol-specific studies, the proportion of respondents found to have recanted has ranged from under 10% (Percy et al. 2005; Shillington and Clapp 2000) to over 30% (Fendrich and Rosenbaum 2003; Shillington et al. 2010). Alcohol-related recanting has tended to be higher amongst males (Percy et al. 2005; Shillington, Clapp, et al., 2011; Siddiqui et al. 1999), although not all studies have confirmed this (Shillington and Clapp 2000), younger respondents (Shillington et al. 2010), ethnic minority respondents (Fendrich and Rosenbaum 2003; Siddiqui et al. 1999), although in some studies, differences due to race/ethnicity have been rather minimal (Shillington and Clapp 2000), respondents with low educational expectations (Percy et al. 2005), and those reporting lower levels of consumption (Fendrich and Mackesy-Amiti 2000).

In one study, young people who reported receiving drug education in the previous 12 months were significantly more likely to recant previous positive reports of cannabis use (Percy et al. 2005). Here, lower reported drug-use behaviours amongst those who received drug education may have been an artefact of increased measurement error (recanting), rather than a reflection of a decrease in actual drug-use behaviour. Similarly, Harris and colleagues observed different reported levels of drug-use inconsistencies across the various arms of a treatment outcome studies (Harris et al. 2008). This opens up the possibility that the positive impact of a prevention intervention may, in part, be due to increased recanting within the intervention arm, relative to the control arm, rather than an actual reduction in consumption (Fendrich 2005; Macleod et al. 2005). This possibility may also help explain the finding that effect sizes are inflated in non-blinded studies with self-report outcomes (Hrobjartsson et al. 2014; Kaner et al. 2017). Given that few universal prevention trials include any non-survey-based confirmation of self-reported substance use, assessing the impact of an identifiable source of measurement error (recanting) on primary outcomes would seem a valuable analytical procedure.

Using data from a large-scale clustered randomised controlled trial (McKay et al. 2018), we aimed to address this gap by examining recanting levels within a school-based alcohol education effectiveness study conducted in the UK. We compared the rates of recanting behaviour across the intervention and control (education as normal) arms and controlled for respondent recanting within the estimation of intervention effects on the trial primary outcomes.

## Method

### Design, Procedures and Sample

The study uses data from a large-scale cluster randomised controlled trial (cRCT) examining the effectiveness of an alcohol education intervention (McKay et al. 2018). A total of 105 post-primary schools, across two geographical locations, participated in the trial: 70 in Northern Ireland (NI) and 35 in Scotland. At the beginning of the study, participants were in their first year of high school (mean age = 12.5 years). Schools were randomly assigned (1:1) to receive the intervention or alcohol education as normal (comprising standard personal, social and health education) before baseline data were collected. Data were collected at baseline (T0) in June 2012 and at three follow-ups: + 12 (T1), + 24 (T2) and + 33 (T3) months. By T3, the mean age of participants was just over 15. The questionnaires were verbally administered to pupils in exam-like conditions. Opt-in consent was obtained from school head-teachers/principals prior to randomisation of the school to either intervention or control. Opt-out consent from participants and their parents/guardians was obtained after randomisation.

The research was approved by Liverpool John Moores University Research Ethics Committee. The trial protocol is available from http://www.nets.nihr.ac.uk/projects/phr/10300209.

### Measures

#### Primary Outcomes

The study had two primary outcomes, both assessed at 33 months. The first was the frequency of self-reported heavy episodic drinking (HED) in the previous 30 days. For male respondents, HED corresponded to consumption of six or more UK units of alcohol in a single session. The corresponding threshold for female respondents was 4.5 units of alcohol. This frequency count was dichotomized at never/one or more occasion. To aid the accuracy of recall, respondents were presented with pictorial prompts of how much alcohol ≥ 6/≥ 4.5 UK units represented. This was a revised primary outcome. This initial proposed HED outcome was the self-reported frequency of consumption of > 5 ‘*drinks’* in a single drinking episode. Concerns arose because it became clear that > 5 ‘drinks’ could refer to drinks of different alcohol strength and volume, and that the intervention had a specific learning outcome around the counting of units consumed. As a result, the HED primary outcome was changed to the ≥ 6/≥ 4.5 UK unit threshold described above. This change was implemented before T3 data collection and before any data was unblinded for analysis. As a result, the indicator of HED employed at T0 (baseline) was the frequency of consuming 5 or more *drinks* in the last month, which was dichotomized.

The second primary outcome was the number of self-reported alcohol-related harms (caused by their own drinking; ARH) in the previous 6 months. Participants were asked to indicate how many times in the past 6 months they had experienced each of 16 separate harms. The frequency count was dichotomised for each harm and then summed across the 16 harms to form an overall count of the number of individual harms experienced ranging from zero to 16.

#### Recanting

At each sweep, respondents were asked if they had ever consumed a ‘full drink’ of alcohol, not just a sip or a taste (yes/no). Recanting was considered to have occurred when a positive lifetime self-report (yes—have consumed a full drink) at one data sweep was followed by a negative lifetime self-report (no—never have consumed a full drink) at a later follow-up. For example, positive lifetime report at T1 followed by a negative lifetime report at T2 would be classified as an incident of recanting at T2. Recanting was coded as a dichotomous indicator (0/1) for each follow-up sweep. An overall recanting indicator was also constructed capturing recanting at any follow-up sweep (T1-T3—again coded 0/1).

### Statistical Analysis

Data cleaning, data management and preliminary analysis were undertaken using IBM SPSS version 22. The primary outcome models were estimated in Mplus 7.11. The outcome analysis was an intention-to-treat (ITT) analysis using the complete case population. Prior sensitivity analysis had suggested that the outcome models were robust to all but extreme missing data assumptions (McKay et al. 2018). A multi-level logistic regression model estimated the association between the intervention and the odds of HED. A multi-level negative binomial regression model estimated the association between the intervention and the number of ARH. All models included school-level random intercepts to account for correlation due to clustering of students within schools. The models also adjusted for factors used to stratify randomisation (location—NI/Scotland; proportion of free school meals—tertile split, low/medium/high), gender of school (in NI only, co-educational/boys only/girls only), the respondents’ recanting (at T3 and at any sweep) and the relevant outcome assessed at baseline. Given that the analysis had two primary outcomes, a statistically significant result was concluded if the *p* value for the trial treatment arm explanatory variable was < 0.025.

## Results

A total of 11,316 pupils participated in the T0 (baseline) data sweep. An additional 1422 pupils who were either absent at T0 but present at T1 data collection (i.e. missing on the day of the T0 data collection) or who joined participating schools between T0 and T1 (before the delivery of phase 1 of the intervention) were also included within the study population. Of the full sample (those who completed a questionnaire at either T0 or T1, *N* = 12,738), 10,405 also completed the questionnaire at T3 (81.7%) and form the complete case population. Table 1 provides the baseline (T0) characteristics of the sample. No differences were detected between the intervention and control arms at T0.

**Table 1:** Demographic characteristics and alcohol outcomes (HED and alcohol-related harms) at baseline (T0) by study arm

	Intervention arm		
	Control (*N* = 5567) *N* (%_column_)	Intervention (*N* = 5749) *N* (%_column_)	Total (*N* = 11,316) *N* (%_column_)
Gender			
Male	2787 (51.1)	2834 (50.0)	5621 (50.5)
Female	2670 (48.9)	2829 (50.0)	5499 (49.5)
Free school meals			
No	4289 (77.3)	4436 (77.5)	8725 (77.4)
Yes	1258 (22.7)	1290 (22.5)	2548 (22.6)
Location			
NI	3469 (62.3)	3554 (61.8)	7022 (62.1)
Scotland	2098 (37.7)	2198 (38.2)	4294 (37.9)
Ethnicity			
White	4492 (95.3)	4495 (94.5)	8987 (94.9)
Non-white	248 (4.5)	293 (5.5)	541 (5.1)
HED			
No	5082 (92.2)	5261 (92.4)	10,343 (92.3)
Yes	432 (7.8)	431 (7.6)	863 (7.7)
Harms (mean ± SD)	0.8 ± 1.9	0.8 ± 2.1	0.8 ± 2.0
Age^a^ (mean ± SD)	12.5 ± 0.4	12.5 ± 0.4	12.5 ± 0.4

Note: ^a^Age was calculated from the pupils’ date of birth to a single time point (1 March 2012). This was initially calculated in days and then divided by 365.25 to give the value in years. *HED*, heavy episodic drinking

At T3, around one in 5 participants reported at least one episode of HED in the last 30 days. The prevalence of HED was nine percentage points higher in the control group (26%) than in the intervention group (17%; odds ratio = 0·60; 95% CI 0·49–0·73) (McKay et al. 2018). Amongst those who had consumed alcohol at T0, around half reported having engaged in HED at T3 within the control schools, compared to just over a third in intervention schools. Around two thirds of pupils (63%) reported no ARH at T3. There was no difference in the number of self-reported ARHs (incident rate ratio = 0·92, CI 0·78–1.05) between control and intervention pupils (McKay et al. 2018).

Around 5% of pupils recanted their alcohol consumption at each data sweep (Table 2). The overall recanting rate across the follow-up period (33 months) was 10%. Recanting was higher amongst males (6% compared to 3%) and respondents in the intervention arm, albeit here, the difference was very small (one percentage point difference). Recanting was lower amongst respondents who reported HED. However, it should be noted that the observed effect sizes, as indexed by Cramer’s *V*, were small to very small. Survey responses were not edited to ensure internal consistency within each sweep. As a result, seven respondents who reported HED at T3 were observed to have also answered ‘never’ to the earlier T3 lifetime use item.

**Table 2:** Proportion of respondents who recanted (T1 to T3) by respondent characteristic

	Recanted at T1%	Recanted at T2%	Recanted at T3%	Any recanting (T1–T3) %	Cramer’s *V* (any recanting)
Arm^a^					
Control	4.5	4.7	4.3**	9.3**	0.02*
Intervention	4.5	5.3	5.3	10.5	
HED (T3)^b^					
No	4.4*	5.7*	7.3**	11.3**	0.07**
Yes	5.6	4.4	0.3	8.7	
Gender^a^					
Male	5.9**	6.3**	6.2**	12.6**	0.09**
Female	3.1	3.7	3.3	7.2	
Free school meals^c^					
Non-eligible	4.6	5.1	4.8	10.1	0.01
Eligible	4.4	5.0	4.5	9.6	
Location^a^					
NI	4.6	5.4*	5.1*	10.2	0.01
Scotland	4.3	4.5	4.2	9.4	

Notes: Seven students who reported HED at T3 also answered that they had never drank alcohol at an earlier survey item. *HED*, heavy episodic drinking. ^a^*N* = 12,738; ^b^*N* = 10,233; ^c^*N* = 12,638; *p < 0.05; **p < 0.01 (chi-square test and Cramer’s *V*)

Initial multi-level ITT outcome analysis indicated that the intervention had a significant impact on HED (Table 3) but no impact on ARH (Table 4) at T3. Although recanting was slightly higher within intervention schools (5.3%) than in control schools (4.3%) and was significantly associated with both primary outcomes (HED and ARH), when entered into the primary outcome models, recanting did not alter the intervention effects observed in the initial outcome analysis. The intervention remained significant in terms of its association with HED (Table 3) and non-significant in its association with ARH (Table 4).

**Table 3:** HED primary outcome analysis unadjusted for recanting (model 1), adjusted for recanting at any sweep (model 2) and adjusted for recanting at T3 (intention-to-treat complete case analysis)

	HED primary outcome model without adjusting for recanting (model 1)				HED primary outcome model adjusting for any recanting (T1–T3) (model 2)				HED primary outcome model adjusting for recanting (T3 only) (model 3)			
	Estimate	CI (95%)		OR	Estimate	CI (95%)		OR	Estimate	CI (95%)		OR
Within school												
Baseline heavy episodic drinking	1.395	1.212,	1.578	4.036	1.472	1.283,	1.661	4.357	1.544	1.347,	1.741	4.683
Recanting (any T1–T3)	–	–	–	–	− 0.491	− 0.691,	− 0.290	0.612	–	–	–	–
Recanting (T3 only)	–	–	–	–	–	–	–	–	− 3.453	− 4.366,	− 2.540	0.032
Between school												
Intervention Arm	− 0.516	− 0.717,	− 0.315		− .508	− 0.710,	− 0.307		− 0.510	− 0.712,	− 0.308	
Free school meals (tertile split)	0.239	0.097,	0.382		0.238	0.095,	0.380		0.249	0.107,	0.390	
School type												
Boy school dummy	− 0.186	− 0.578,	0.205		− 0.209	− 0.606,	0.187		− 0.226	− 0.621,	0.168	
Girl school dummy	− 0.546	− 1.068,	− 0.025		− 0.539	− 1.058,	− 0.020		− 0.522	− 1.019,	− 0.026	
Location (NI)	0.422	0.209,	0.635		0.419	0.205,	0.633		0.416	− 0.199,	0.632	
Residual variances	0.176	0.107,	0.244		0.178	0.109,	0.247		0.176	0.109,	0.244	
Threshold (BngT3$1)	1.574	1.333,	1.819		1.534	1.289,	1.739		1.512	1.268,	1.755	

Note. The logistic regression multi-level models were estimated using a logit link function and the maximum likelihood estimation with robust standard errors (MLR) estimator. *HED*, heavy episodic drinking

**Table 4:** Alcohol-related harms (ARH) primary outcome analysis unadjusted for recanting (model 1), adjusted for recanting at any sweep (model 2) and adjusted for recanting at T3 (intention-to-treat complete case analysis)

	ARH primary outcome model without adjusting for recanting (model 1)			ARH primary outcome model adjusting for any recanting (T1–T3) (model 2)			ARH primary outcome model adjusting for recanting (T3 only) (model 3)		
	Estimate	CI (95%)		Estimate	CI (95%)		Estimate	CI (95%)	
Within school									
Baseline alcohol-related harms	0.211	0.189,	0.232	0.215	0.192,	0.237	0.215	0.193,	0.238
Recanting (any T1–T3)	–	–	–	− 0.353	− 0.496,	−0.210	–	–	–
Recanting (T3 only)	–	–	–	–	–	–	− 2.587	− 2.955,	− 2.218
Between school									
Intervention arm	− 0.101	− 0.264,	0.061	− 0.097	− 0.261,	0.066	− 0.092	− 0.253,	0.069
Free school meals (tertile split)	0.168	0.049,	0.287	0.164	0.046,	0.282	0.170	0.055,	0.285
School type									
Boy school dummy	− 0.083	− 0.483,	0.317	− 0.091	− 0.495,	0.314	− 0.120	− 0.519,	0.279
Girl school dummy	− 0.380	− 0.843,	0.082	− 0.379	− 0.834,	0.077	− 0.336	− 0.760,	0.089
Location (NI)	0.433	0.273,	0.593	0.425	0.264,	0.586	0.418	0.257,	0.579
Residual variances	0.115	0.063,	0.167	0.117	0.066,	0.169	0.116	0.067,	0.164
Intercept (harms T3)	− 0.042	− 0.225,	0.140	− 0.009	− 0.164,	0.176	0.008	− 0.178,	0.188
Dispersion (harms T3)	3.563	3.158,	3.968	3.538	3.132,	3.944	3.306	2.924,	3.688

Note: The models estimated assumed a negative binomial distributed count variable and employed a MLR estimator. Models assuming a Poisson distribution were also estimated. However, the negative binomial models had a better fit (lower AIC) and a significant dispersion parameter. *N* = 10,380

## Discussion

This study examined the extent of recanting within a large school-based alcohol prevention trial. By replication of the original trial primary outcome analysis, but this time controlling of instances of recanting (both any recanting and recanting at the final follow-up), the study also assessed the impact on recanting on the estimation of the intervention effect.

Results of this study revealed that recanting is a noticeable source of measurement error within school-based alcohol trials, involving around one in 20 respondents each data sweep, and a source of measurement error that is associated with traditional consumption-based outcomes measures. The level of recanting estimated within this study was at the lower range of previous estimates (Percy et al. 2005; Shillington and Clapp 2000) and confirmed prior reports of inflated recanting amongst male students (Percy et al. 2005; Siddiqui et al. 1999).

In terms of between-group differences, there was a differential rate of recanting across the study arms (intervention versus control) and across gender, although the effect of recanting was very small, as evidenced by the Cramer’s *V* values. While recanting was higher amongst pupils who received a harm reduction alcohol education intervention than those who received education as normal, the observed difference was only a single percentage point difference, compared to the nine percentage point difference observed in primary outcome HED. Based on this finding, recanting may not be the predominant cause of inflated effect sizes in non-blinded studies with self-report alcohol outcomes (Hrobjartsson et al. 2014; Kaner et al. 2017), although further replication is required before a robust conclusion can be drawn.

Recanting arises from a lack of consistency in reporting across survey sweeps. This inconsistency may be due to deliberate attempts to misreport consumption (under-reporting following a positive report) or a failure to maintain a deception over multiple survey sweeps (an over-report that is not repeated in subsequent sweeps) (McCambridge and Strang 2006). Either way, the incident of recanting indicates a false report at one or more data sweeps. It is also possible that recanting arises due to simple errors in answering the pencil and paper questionnaire (ticking no when they meant to tick yes), rather than deliberate attempts to misreport consumption. However, as only seven students who recanted lifetime alcohol use went on to report HED, this may not be a significant issue in a study such as this. Those who did recant appeared to be consistent in their reports of alcohol consumption within a survey sweep, even when inconsistent between sweeps.

While the analysis has a number of methodological strengths including a large sample size, robust randomisation and analysis that accounted for the multi-level structure of the data collected, it does have some limitations. Firstly, while the study identified incidents of recanting, it was unable to determine whether these were the result of either initial over-reporting (i.e. a false initial claim of lifetime use that was corrected at follow-up) or later under-reporting (a true initial claim of lifetime use that was denied at follow-up). It is likely that these distinct forms of self-report inconsistencies will have a different impact on the assessment of trial outcomes. Secondly, the study did not incorporate any non-questionnaire-based self-report measures (Koning et al. 2010) or non-survey-based measures of consumption, although these are likely to be non-viable in large-scale surveys (Taylor et al. 2017). As a result, it had no independent corroboration on reporting inconsistencies. And finally, the study was only able to assess the impact of recanting on alcohol outcomes. The impact of recanting may be greater for more socially undesirable adolescent behaviours (i.e. cannabis and other drug use) (Fendrich and Mackesy-Amiti 1995; Fendrich and Rosenbaum 2003; Harris et al. 2008; Johnston and O'Malley 1997; Percy et al. 2005).

In conclusion, this study demonstrates, for what we believe may be first time, that recanting is an important form of measurement error within in school-based prevention RCTs, but one that is readily identifiable and easily controlled for within RCT outcome analysis, using the method demonstrated above. Recanting varies with known predictors of alcohol consumption (e.g., gender) (Chassin et al. 2002) and with standard alcohol outcome measures routinely used in prevention RCTs. We recommend including recanted self-reports as a covariate within planned outcome analysis as a simple method of adjusting intervention effects for a known source of self-report measurement error associated with consumption-based outcomes. Another option would be test different recanting assumptions within any outcome analysis (i.e. using a worst case scenario—setting all who recant to HED; a best case scenario—setting all who recant to non-HED; or a conservative case scenario—setting all who recant in the treatment arm to HED and all who recant in the control arm to non-HED) to assess the sensitivity of the outcome results to variations in those assumptions, as is routinely done with missing data (see for example McKay et al. 2018).

While the levels of recanting were low within this study, this may not always be the case, particularly for non-alcohol substance use outcomes where social desirability pressures are considerably greater (Shillington et al. 2010). Therefore, we recommend that recanting analysis should be readily incorporated with the broader suite of sensitivity tests undertaken to assess other forms of error within RCTs, such as those for missing data and alternative specifications of the trial primary outcomes (Thabane et al. 2013).
